# TRAF-6 Dependent Signaling Pathway Is Essential for TNF-Related Apoptosis-Inducing Ligand (TRAIL) Induces Osteoclast Differentiation

**DOI:** 10.1371/journal.pone.0038048

**Published:** 2012-06-14

**Authors:** Men-Luh Yen, Ping-Ning Hsu, Hsiu-Jung Liao, Be-Hang Lee, Hwei-Fang Tsai

**Affiliations:** 1 Department of General Medicine, College of Medicine, National Taiwan University, Taipei, Taiwan; 2 Department of Obstetrics and Gynecology, National Taiwan University Hospital, Taipei, Taiwan; 3 Graduate Institute of Immunology, College of Medicine, National Taiwan University, Taipei, Taiwan; 4 Department of Internal Medicine, National Taiwan University Hospital, Taipei, Taiwan; 5 Department of Internal Medicine, Taipei Medical University Shuang Ho Hospital, Taipei, Taiwan; 6 Graduate Institute of Clinical Medicine, College of Medicine, Taipei Medical University, Taipei, Taiwan; Mayo Clinic, United States of America

## Abstract

Human osteoclast formation from mononuclear phagocyte precursors involves interactions between tumor necrosis factor (TNF) ligand superfamily members and their receptors. Recent evidence indicates that in addition to triggering apoptosis, the TNF-related apoptosis-inducing ligand (TRAIL) induces osteoclast differentiation. To understand TRAIL-mediated signal transduction mechanism in osteoclastogenesis, we demonstrated that TRAIL induces osteoclast differentiation via a Tumor necrosis factor receptor-associated factor 6 (TRAF-6)-dependent signaling pathway. TRAIL-induced osteoclast differentiation was significantly inhibited by treatment with TRAF-6 siRNA and TRAF6 decoy peptides in both human monocytes and murine RAW264.7 macrophage cell lines, as evaluated in terms of tartrate-resistant acid phosphatase (TRAP)-positive multinucleated cells and bone resorption activity. Moreover, TRAIL-induced osteoclast differentiation was also abolished in TRAF6 knockout bone marrow macrophages. In addition to induction of NFATc1, treatment of TRAIL also induced ubiquitination of TRAF6 in osteoclast differentiation. Thus, our data demonstrate that TRAIL induces osteoclastic differentiation via a TRAF-6 dependent signaling pathway. This study suggests TRAF6-dependent signaling may be a central pathway in osteoclast differentiation, and that TNF superfamily molecules other than RANKL may modify RANK signaling by interaction with TRAF6-associated signaling.

## Introduction

Osteoclasts are multinucleated cells, derived from precursors of monocyte/macrophage lineages. Osteoclasts are involved in bone absorption and remodeling. It is already known that normal differentiation of osteoclasts requires TNF family receptors, such as the receptor activator of nuclear factor-κB (RANK) [1], [2], [3], [4], [5]. It is likely that the RANK/RANK ligand (RANKL)/osteoprotegerin (OPG) system system is the central and primary regulator of bone remodeling; however, it is clear that this is not the only mechanism involved. Many of the cytokines and growth factors implicated in inflammatory processes in rheumatic diseases have also been demonstrated to impact osteoclast differentiation and function either directly, by acting on cells of the osteoclast-lineage, or indirectly, by acting on other cell types to modulate expression of the key osteoclastogenic factor, RANKL, and/or its inhibitor, OPG [6], [7], [8], [9], [10]. In addition to RANKL, recent studies have demonstrated there are several TNF family molecules which promote osteoclast differentiation, including TNF [11], decoy receptor 3 (DcR3) [12], FasL [13] and TRAIL [14]; indicating that activated T cells and inflammatory response can remodel bone homeostasis via these effector molecules. TRAIL, a member of the TNF ligand superfamily, induces apoptosis in diverse tumor cell lines [15], and its expression is upregulated in activated T cells. In our previous studies, we have demonstrated that in addition to triggering apoptosis, TRAIL induces osteoclast differentiation in mononuclear phagocyte precursors [14]. Our results indicate that this mechanism may be implicated in osteoimmunology in immune response-associated bone absorption. However, the mechanism and signaling pathways of TRAIL-induced osteoclast differentiation is still not clear. Ligands for these receptors plus unidentified serum or cell-presented factor(s) are necessary for *in vitro* differentiation, indicating the involvement of signaling pathways possibly via an immune-like tyrosine kinase acceptor molecule. RANK provokes biochemical signaling via the recruitment of intracellular tumor necrosis factor receptor-associated factors (TRAFs) after ligand binding and receptor oligomerization. Accumulating evidence from various laboratories indicates TRAFs, most importantly TRAF6, is the key to understanding how RANKL links cytoplasmic signaling to the nuclear transcriptional program [16], [17], [18], [19], [20]. However, the signaling pathways for TRAIL-induced osteoclast differentiation and whether TRAF6-dependent signaling is essential for this effect is still not clear. To understand the TRAIL-mediated signal transduction mechanism in osteoclastogenesis, we study role of TRAF6 –dependent signaling in TRAIL-induced osteoclast differentiation and bone resorption. Our results indicate that TRAF6 is essential for TRAIL-induced osteoclast differentiation and bone resorption activity. This study suggests TRAF6-dependent signaling may be a central pathway in osteoclast differentiation, and TNFs other than RANKL may modify RANK signaling by interaction with TRAF6-associated signaling.

**Figure 1:**
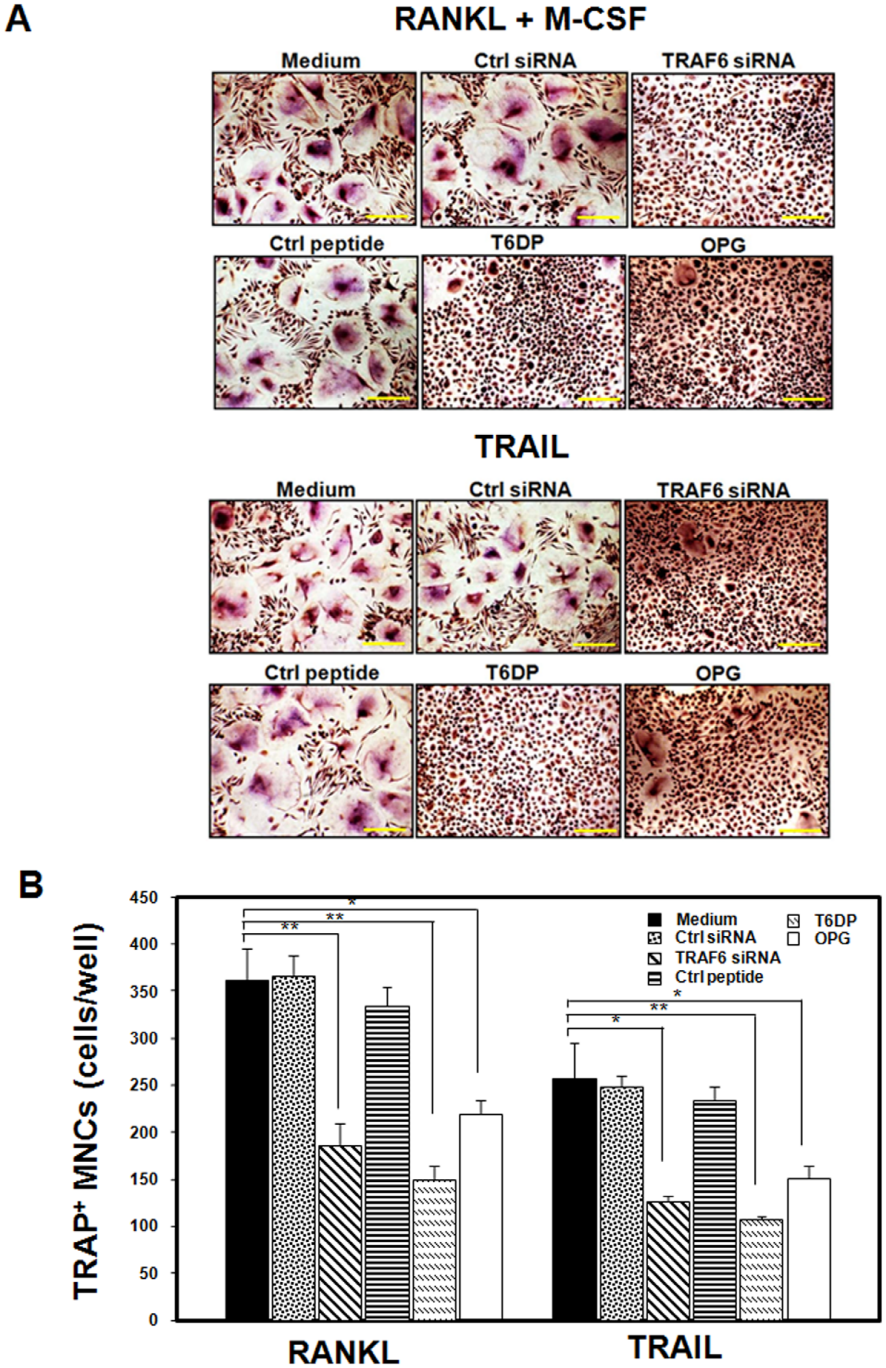
TRAIL induced osteoclast differentiation is dependent on TRAF6. (A) Human peripheral blood mononuclear cells (PBMCs) were plated in 96-well plates at 1.5 × 10^5^ cells/well, and the next day the adherent monocytes were treated with TRAIL (500 ng/ml) as well as M-CSF (50 ng/ml) and RANKL (100 ng/ml) in the presence or absence of TRAF6 siRNA, control siRNA (Crtl siRNA), TRAF6 decoy peptide (T6DP), control peptide (Crtl peptide), or osteoprotegerin (OPG) as indicated in the figure for 30 days. The transfection of siRNAs into human macrophages cells was performed by electroporation. The TRAF6 inhibitory peptide, T6PD and its control peptide were used to examine its effects on TRAIL-mediated osteoclast differentiation. After incubation, cells were subjected to the TRAP assay. Cell morphology was examined by light microscopy, and the number of TRAP-positive multinuclear cells was quantified. The bar in each figure represents 100 µM. (B). TRAIL-induced formation of osteoclast-like multinucleated cells from human monocytes. Data in (B) represent the mean±SD from three to six independent experiments. *p < 0.05, **p < 0.005.

## Materials and Methods

### Cell Lines

We used human peripheral blood mononuclear cells (PBMCs) and the RAW264.7 murine monocytic/macrophagic cell line as model systems of osteoclastogenesis. Both cell types differentiate into osteoclast-like cells in the presence of RANKL plus M-CSF [21], [22]. RAW264.7 cells, which were derived from a tumor induced by the Abelson murine leukemia virus, were purchased from American Type Culture Collection (ATCC; Rockville, MD). Cells were cultured in Dulbecco's modified Eagle's medium (DMEM) supplemented with 10% fetal calf serum (FCS), 2 nM L-glutamine, and 100 U/ml penicillin/streptomycin. For osteoclast culture, RAW cells were seeded in a 96-well plate at a density of 2×10^3^ cells/well and cultured for 5 days in the presence of 25 ng/ml M-CSF and 50 ng/ml RANKL, as the positive control, or 0.5 µg/ml TRAIL. The medium was replaced every 3 days. Human PBMCs from healthy donors were separated by gradient centrifugation with Ficoll-Hypaque and were re-suspended in α-MEM supplemented with 10% heat-inactivated FBS. PBMCs were then plated in 96-well plates at 1×10^6^ cells/well and incubated in the absence or presence of 500 ng/ml TRAIL for 30 days. The medium was replaced every 3 days.

**Figure 2:**
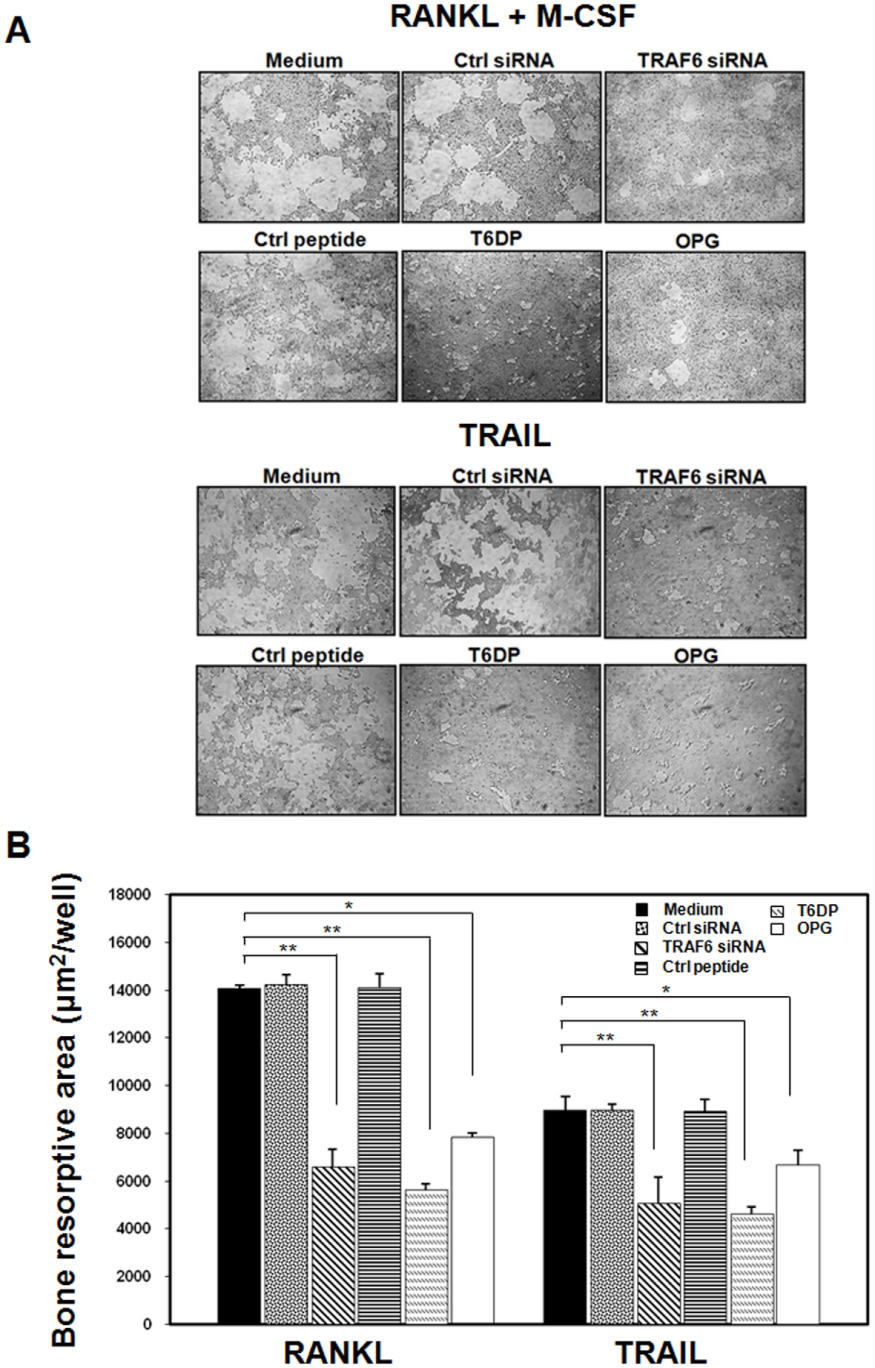
TRAIL-induced osteoclast bone resorption activity is TRAF6-dependent. (A). Human monocytes were plated on an artificial bone matrix slide (calcium phosphate apatite-coated plate; OAAS) and were cultured with TRAIL (500 ng/ml) as well as M-CSF (50 ng/ml) and RANKL (100 ng/ml) in the presence or absence of TRAF6 siRNA, control siRNA (Crtl siRNA), TRAF6 decoy peptide (T6DP), control peptide (Crtl peptide), or osteoprotegerin (OPG) as indicated. Cells were detached after 30 days of culture. The number of resorption pits and resorptive area in each well was observed and counted using a microscope. (B). The resorption pits were photographed and the resorptive area in each well was counted. Data in (B) represent the mean±SD from five independent experiments. *p < 0.05, **p < 0.005.

### Transfection of TRAF6 or TRAIL-R siRNAs

To test the role of TRAF6 or TRAIL-R in TRAIL-induced osteoclastogenesis, RAW 264.7 cells were transfected with a pool of 4 specific siRNAs (Thermo Scientific, Dharmacon, CO) or nonspecific control siRNA, and then treated with TRAIL. The ON-TARGET plus SMART pool TRAF6 siRNA target sequences were GCACAGCAGUGUAACGGGA, GGACAAGGUUGCCGAAAUG, GAGAACAGAUGCCUAAUCA, GCUCAAUCGUUUAAUAAGA. The ON-TARGET plus SMART pool TRAIL-R siRNA target sequences were UAAGGAAGAUAAAGUCGUA, UAAGCUAGGCCUCUGGAUA, GAAACCCGAUGCAACAUAA, GAGCAUGGAGGCAAUGGUU.

**Figure 3:**
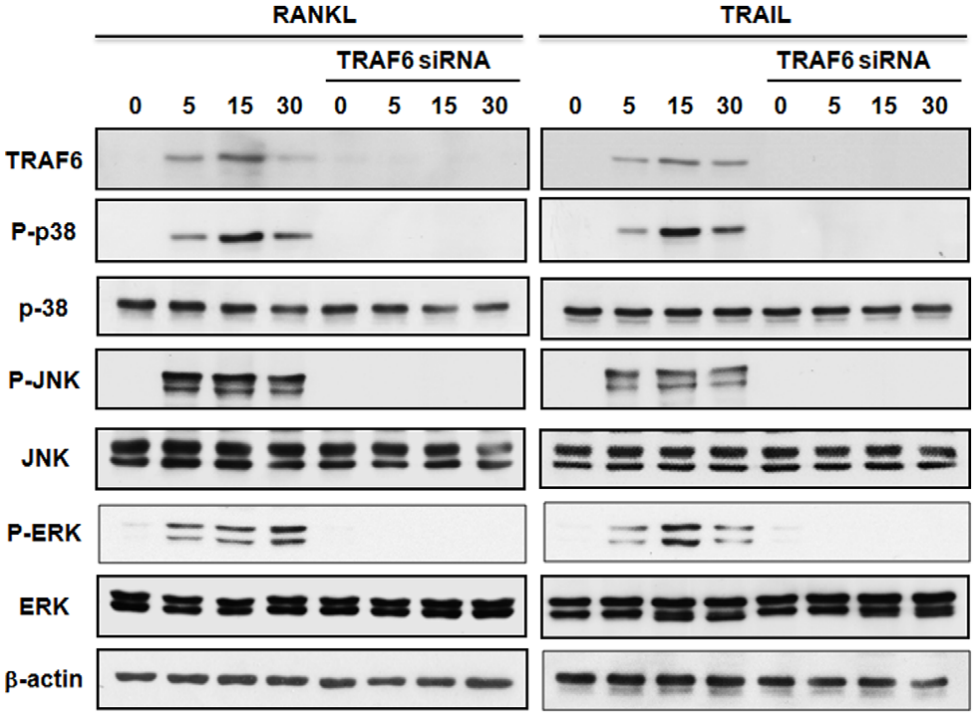
TRAIL induces activation of MAP Kinases is dependent on TRAF6. RAW264.7 cells were treated with TRAIL (500 ng/ml), M-CSF (20 ng/ml) and RANKL (50 ng/ml) in the presence or absence of TRAF6 siRNA. After stimulation, cells were solubilized, and cell lysates were subjected to Western blot analysis of TRAF6, p38 MAPK, JNK, and ERK1/2. The trace shown in the top panel for each group indicates the immunoreactivity of the phosphorylated kinase. The same membrane was then stripped and reprobed with the kinase antibody recognizing the total protein level of kinase (bottom panel). The results are representative of five separate experiments.

The transfection of siRNA into human macrophages and RAW 264.7 cells was performed by electroporation. In brief, RAW 264.7 cells (5×10^6^ cells) were gently mixed with siRNA (2nmole) in electroporation buffer (0.8% glucose, 20% FBS in antibiotics-free RPMI), transferred to an electroporation cuvette, and electroporated using the GenepluserII device. The expression of TRAIL-R was detected with anti-TRAIL-R mAb (Chemicon and Upstate, Billerica, MA).

**Figure 4:**
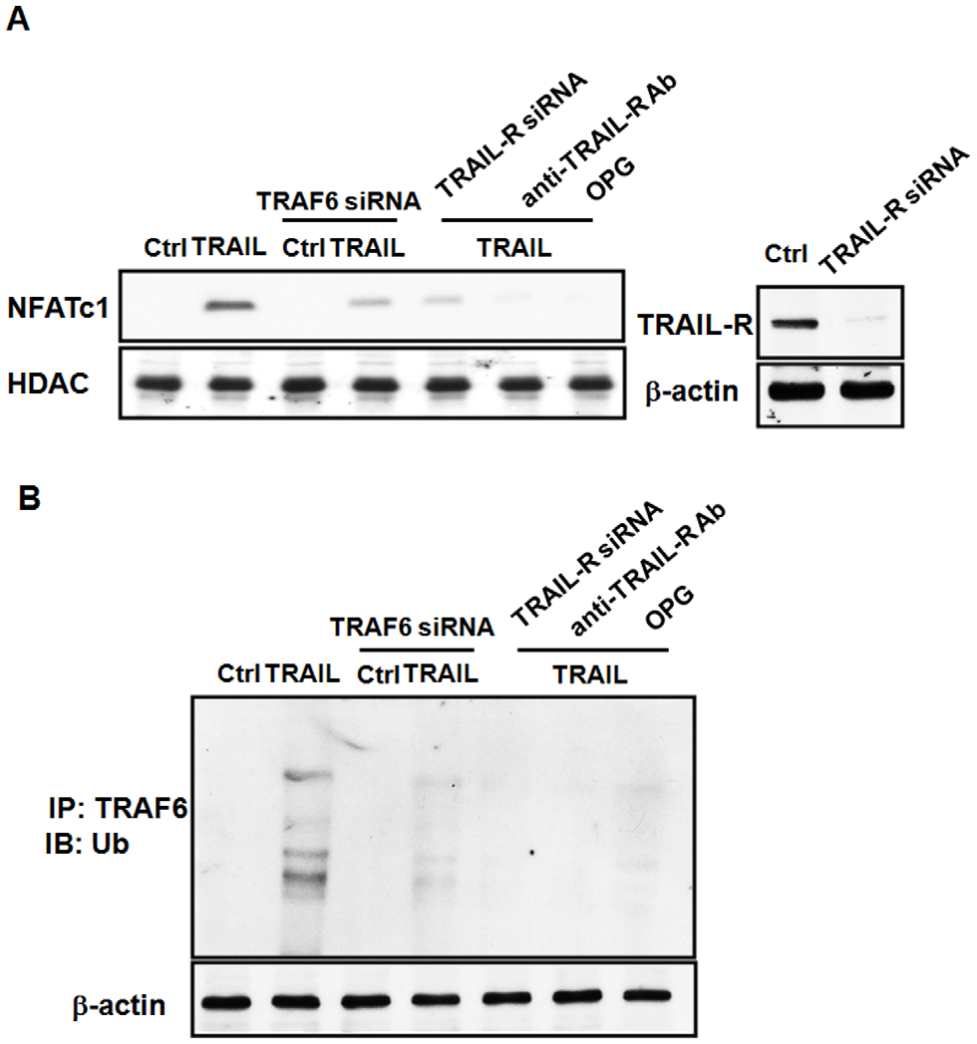
TRAIL-induced activation of NFATc1 in osteoclast differentiation is dependent on TRAF6. (A). RAW264.7 cells were treated with TRAIL, M-CSF (20 ng/ml) and RANKL (50 ng/ml) in the presence or absence of TRAF6 siRNA, TRAIL receptor (TRAIL-R) siRNA, anti-TRAIL-R antagonist Ab, or OPG as indicated in the figure. After stimulation, cells lysates of the nuclear fraction were prepared, and immunoblotted with anti-NFATc1 and anti-HDAC antibodies. The results are representative of three separate experiments. (B).TRAF6 is ubiquitinated after TRAIL stimulation. Ubiquitin transfected RAW 264.7 cells were treated with TRAIL in the presence or absence of TRAF6 siRNA, TRAIL-R siRNA, anti-TRAIL-R antagonist Ab, or OPG as indicated in the figure. The cell lyses were immunoprecipitated with anti-TRAF6 mAb. Bound proteins were subjected to SDS–PAGE and immunoblotted with anti-Ubiquitin mAb, and then the membrane was stripped and reprobed with anti-TRAF6 mAb. The results are representative of three separate experiments.

### Osteoclast Differentiation

For osteoclastic differentiation, cells were cultured in the presence of either 50 ng/mL human M-CSF (for PBMCs; R&D Systems, Minneapolis, MN) or murine M-CSF (for RAW264.7; R&D Systems), and 50 ng/mL human RANKL (for PBMCs, R&D Systems) or murine RANKL (for RAW264.7, R&D Systems), or recombinant TRAIL proteins purified from an *Escherichia coli* expression system as previously described [23]. To remove the endotoxin, the purified recombinant TRAIL proteins were further treated with Detoxi-GelTM Endotoxin Removing Gel (Pierce, Rockford, IL) to remove the lipopolysaccharide (LPS) during purification. The absence of endotoxin contamination in the recombinant TRAIL preparation (<0.1 endotoxin units/mL) was assessed by a Limulus Amoebocyte Lysate (LAL) assay (BioWhittaker, Walkersville, MD).

**Figure 5:**
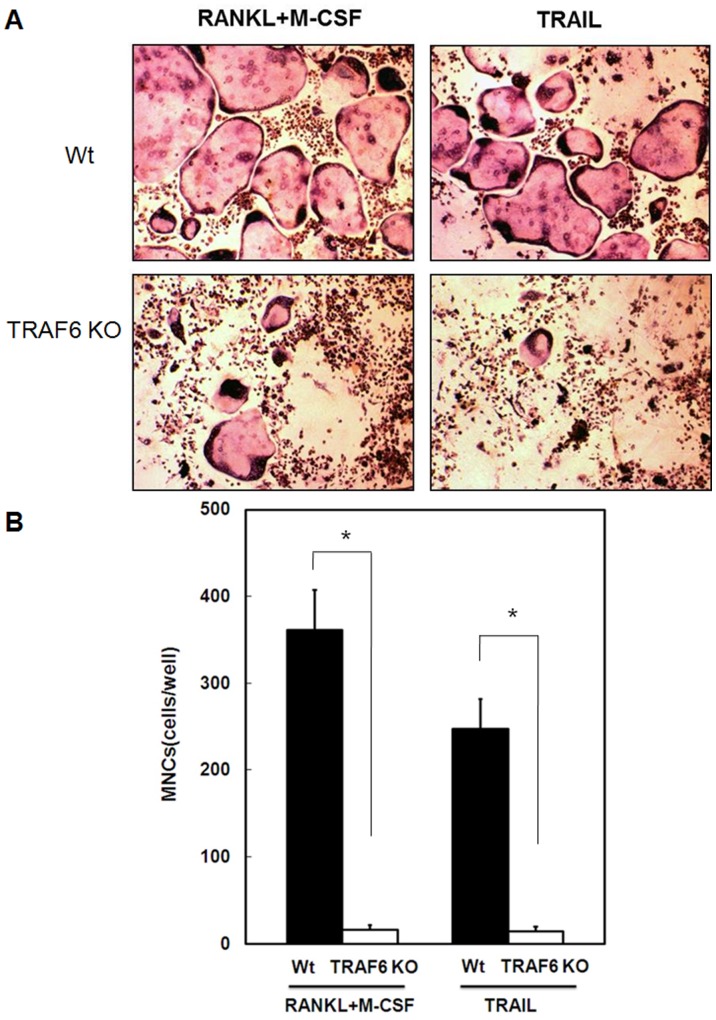
TRAIL-induced osteoclast differentiation was abolished in TRAF6 knock out bone marrow-derived macrophages. (A) Bone marrow-derived macrophages isolated from TRAF6 knock out (TRAF6 KO) mice were treated with TRAIL (500 ng/ml) or RANKL (100 ng/ml) with M-CSF (200 ng/ml) for 7 days. After incubation, cells were subjected to the TRAP assay. Cell morphology was examined by light microscopy, and the number of TRAP-positive multinuclear cells was quantified. (B). TRAIL-induced formation of osteoclast-like multinucleated cells from bone marrow-derived macrophages isolated from wild type and TRAF6 KO mice. Data in (B) represent the mean±SD from three independent experiments. *p < 0.005.

The TRAF6 inhibitory peptide, T6PD (DRQIKIWFQNRRMKWKKRKIPTEDEY; IMGENEX, San Diego, CA), which was shown to specifically target TRAF6 and inhibit RANKL-mediated signaling and osteoclast differentiation [24], was used to examine its effects on TRAIL-mediated osteoclast differentiation in RAW macrophages. For some experiments, we also used recombinant osteoprotegerin (OPG) Fc fusion protein (R&D System Inc, Minneapoli, MN) and anti-TRAIL-R blocking antibody (R&D System Inc, Minneapolis, MN) to block the interaction between TRAIL and TRAIL receptor in osteoclast differentiation.

**Figure 6:**
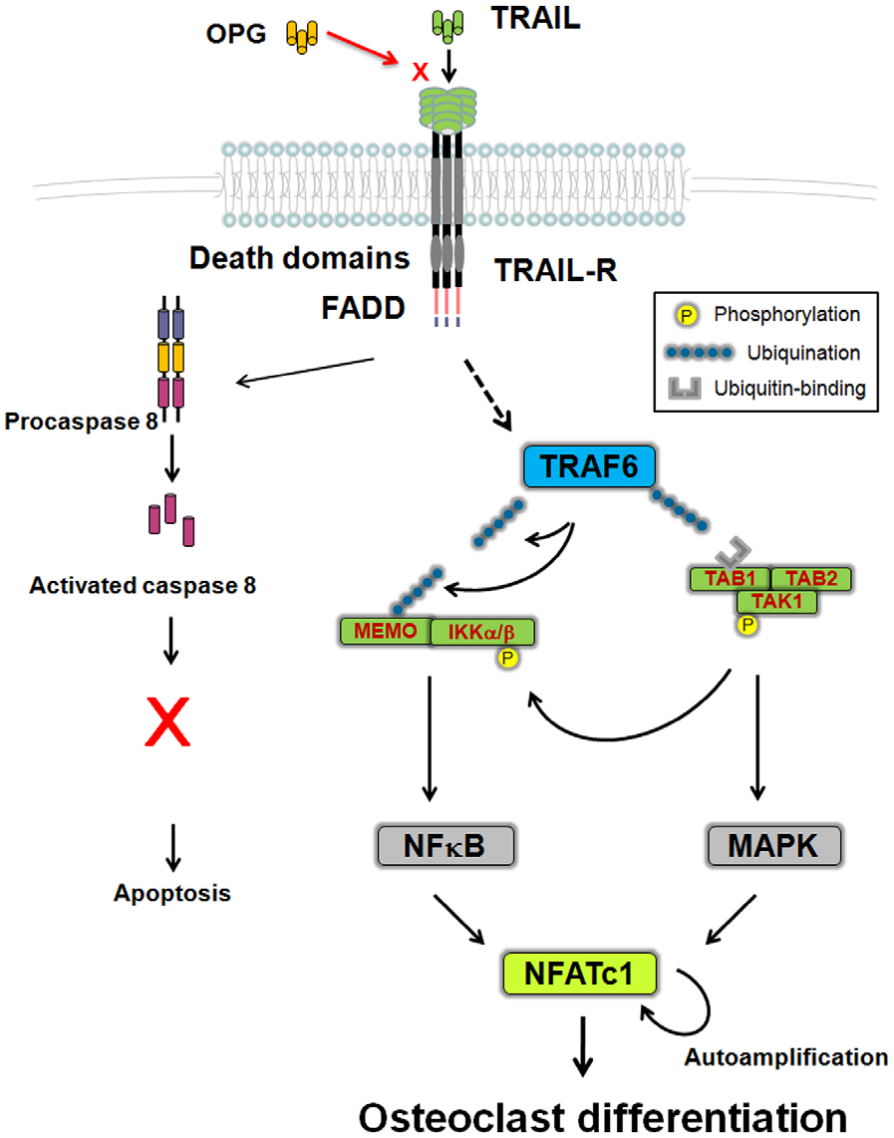
A model for TRAIL-induced activation of the TRAF6 associated signaling pathway in osteolcast differentiation. Upon receptor engagement, in addition to transduce apoptosis signaling pathway, TRAIL induces activation of TRAF6, at least in part through the E3 ubiquitin ligase activity of the TRAF6 RING finger domain via a distinct signaling pathway. In osteoclast precursors, TRAIL is not able to induce sufficient amount of signals to activate the downstream apoptosis signaling to induce apoptosis; instead, TRAIL induces signaling to activate TRAF6 after receptor engagement. TRAF6 is the key molecule linking cytoplasmic signaling to the nuclear transcriptional program in osteoclast differentiation and bone resorption. These signals include activation of NF-κB by a process of ubiquitination in which TRAF6 functions as an E3 ligase [36] and recruitment of the TAB1-TAB2-TAK1 complex, which lead to the osteoclast-specific event, that is, autoamplification of NFATc1, the master transcription factor for osteoclast differentiation [29], [30], [31].

### TRAP Staining

Osteoclast formation was measured by quantifying cells positively stained with TRAP (Acid Phosphatase Kit 387-A; Sigma-Aldrich, St. Louis, MO). Briefly, specimens were fixed for 30 s and then stained with naphthol AS-BI phosphate and a tartrate solution for 1 h at 37°C, followed by counterstaining with a hematoxylin solution. Osteoclasts were determined to be TRAP-positive staining multinuclear (>3 nuclei) cells using light microscopy. TRAP-positive multinuclear cells with three or more nuclei were regarded as osteoclasts and counted under an inverted-phase contrast microscope. The total number of TRAP-positive cells and the number of nuclei per TRAP-positive cells in each well were counted. The morphological features of osteoclasts were also photographed.

### Bone Resorption Assay

In order to confirm the bone resorption ability by differentiated osteoclasts, human monocytes were plated onto a 24-well plates coated with artificial bone slides (calcium phosphate apatite plate; OAAS, Osteogenic Core Technologies, Irvine, CA) under the same culture conditions as described above. After a 30- day culture, each well was washed with saline, and a solution of 5% sodium hypochlorite was left in the well for 5 min in order to detach the cells. The number of resorption pits and bone resorptive area in each well was counted under a microscope. The pits were also photographed.

### Flow Cytometric Analysis

For the flow cytometric analysis, adherent cells were harvested from culture plates by gentle scraping on ice. Surface cell staining was performed at 4°C for 40 min by incubating 3 × 10^5^ cells in 200 µL phosphate-buffered saline (PBS; containing 1% bovine serum albumin (BSA) and 5% human plasma) with the indicated antibodies (Abs). Expression of monocytic/macrophagic markers was documented using phycoerythrin (PE)-conjugated anti-CD14 (Immunotech, Marseille, France) and anti-CD36 monoclonal Abs (mAbs; BD Pharmingen, San Diego, CA), and fluorescein isothiocyanate (FITC)-conjugated anti-CD64 mAb (Immunotech). Nonspecific fluorescence was assessed by incubation with irrelevant isotype-matched conjugated mAbs. Flow cytometric analyses were performed by FACScan (Becton Dickinson, San Diego, CA).

### Activation of NFATc1

For detecting activation of NFATc1, the RAW264.7 cells were treated with TRAIL (500 ng/ml), M-CSF (20 ng/ml) and RANKL (50 ng/ml) in the presence or absence of TRAF6 siRNA. After stimulation, cells lysates of the nuclear fraction were prepared. Nuclear and cytosolic proteins were separated upon resuspension of the pelleted cells (10^6^) in 30 µl hypotonic lysis buffer [10 mM Tris-HCl (pH 7.4), 10 mM NaCl, 3 mM MgCl_2_, and 0.5% NP-40] by gentle pipetting, then incubation on ice for 5 min. The homogenate was centrifuged at 6000 rpm for 5 min at 4°C. The supernatant representing the cytosolic fraction was collected then stored at –80°C, and the pellet containing cell nuclei was lysed in 30 µl RIPA lysis buffer. The nuclear extract (supernatant) was retained after centrifugation at 13,000 g for 5 min at 4°C for subsequent Western blotting with anti-NFATc1 (Thermo Scientific, Rockford, IL) and anti-HDAC antibodies (Cell signaling, Danvers, MA).

### Ubiquitination of TRAF6

For assaying ubiquitination of TRAF6, we transfected the ubiquitin plasmid into RAW 264.7 cells, and the cells were treated with recombinant TRAIL. The whole-cell lysate was prepared 24 h after treatment with TRAIL. The cell lysates were then Immunoprecipitated with an anti-TRAF6 polyclonal antibody (Santa Cruz Biotechnology, Santa Cruz, CA), and analyzed by SDS-PAGE and Western blotting transferred to nitrocellulose membranes. TRAF6 ubiquitination was detected with an anti-ubiquitin antibody (Santa Cruz Biotechnology, Santa Cruz, CA) and anti-TRAF6 mAb (Abcam, Cambridge, UK). The ubiquitin plasmid was kindly provided from Dr. Ming-Zong Lai (Academia sinica, Taiwan).

### Statistical Analysis

Data are expressed as the mean±SD from at least three experiments. Student's *t*-test was used to assess the statistical significance of the differences, and a *p* value of <0.05 was considered statistically significant.

## Results

### TRAIL Induced Osteoclast Differentiation in Human PBMCs and Murine RAW264.7 Cells is TRAF6 Dependent

To determine role of TRAF6 –dependent signaling in TRAIL-induced osteoclast differentiation, we first investigated the influence of TRAF6 in TRAIL-induced osteoclast differentiation in human preosteoclastic precursors. For this purpose, human PBMCs were allowed to adhere for 3∼5 days. The adherent PBMCs were positive for the monocytic/macrophagic markers CD14, CD36, and CD64 at this time point (data not shown). Monocyte precursors can differentiate into osteoclast-like cells in the presence of RANKL (100 ng/ml) and M-CSF (50 ng/ml) or TRAIL (500 ng/ml). This differentiation was verified not only by the appearance of multinuclear cells but also by the positive staining with TRAP. The formation of large, multinucleated TRAP^+^ cells was observed, characterized by the appearance of TRAP-positive giant multinucleated cells with more than three nuclei. In our previous study, TRAIL in this does range did not induce apoptosis; instead, TRAIL induced osteoclast differentiation in preosteoclastic precursors [14] (Figure S1). We then examined whether the TRAIL-induced osteoclast differentiation activity is dependent on TRAF6 signaling pathway after TRAIL engagement. The monocyte precursors were treated with TRAIL (500 ng/ml) as well as M-CSF (50 ng/ml) and RANKL (100 ng/ml) in the presence or absence of TRAF6 siRNA, TRAF6 decoy peptide (T6DP) or osteoprotegerin (OPG). In addition to being an inhibitor of RANKL, OPG is also a decoy receptor for the cytotoxic ligand TRAIL [25]. OPG can bind to TRAIL to neutralize the osteoclast differentiation effects induced by TRAIL [14]. In Figure 1, our results demonstrated that both RANKL- and TRAIL-induced osteoclast differentiation ability was significantly inhibited after silencing the expression of TRAF6 by using TRAF6 siRNA, indicating that TRAIL-induced osteoclast differentiation activity is dependent on TRAF6 (Figure 1). Both RANKL- and TRAIL-induced osteoclast differentiation was also significantly reduced when treated with OPG.

For further demonstration of the role of TRAF6 in TRAIL-mediated signaling in osteoclast differentiation, a cell-permeable TRAF6 decoy peptide (T6DP), which was shown to specifically target TRAF6 and inhibit RANKL-mediated signaling and osteoclast differentiation [24], was used to examine its effects on TRAIL-mediated osteoclast differentiation in human macrophages. The results in Figure 1 demonstrated that TRAIL-induced osteoclast differentiation was significantly reduced after adding the TRAF6 decoy peptide, T6DP into the culture, indicating that TRAIL-induced osteoclast differentiation activity is dependent on TRAF6 signaling pathway.

### TRAIL-induced Osteoclast Differentiation and Bone Resorption Activity is Dependent on TRAF6

To further determine whether TRAF6 signaling pathway is essential for TRAIL-induced osteoclast differentiation and bone resorption activity, we performed functional assays for identification of osteoclast differentiation in an *in vitro* culture system. Using commercial calcium phosphate apatite as a resorption substrate, pit formation caused by TRAIL-treated human macrophages was compared to cultures of cells in the presence or absence of TRAF6 siRNA and the TRAF6 decoy peptide, T6DP (Figure 2). Lacunar resorption was observed in human macrophages treated with TRAIL on calcium phosphate apatite plates; however, the bone resoprtion activity was significantly reduced after treatment with TRAF6 siRNA or the TRAF6 decoy peptide, T6DP (Figure 2). Taken together, our results clearly demonstrated that TRAF6 is essential for TRAIL-induced osteoclast differentiation and bone resorption activity.

### TRAIL-induced Activation of MAP Kinases is Dependent on TRAF6

Three members of MAPKs, ERK, p38 MAPK, and JNK, have been implicated in the mediation of cytokine-regulated osteoclastogenesis [26], [27], [28]. To elucidate the signaling pathways underlying TRAIL's action, we examined the activation of MAPKs in RAW264.7 cells treated with TRAIL, RANKL and M-CSF in the presence or absence of TRAF6 siRNA by immunoblotting. As shown in Figure 3, expression of TRAF6 was upregulated when treated with TRAIL or RANKL plus M-CSF in RAW264.7 macrophages. TRAIL induced the phosphorylation of p38 MAPK, JNK, and ERK in osteoclast precursors, and silencing of TRAF6 by treatment of TRAF6 siRNA inhibited the TRAIL-induced phosphorylation of p38 MAPK, JNK and ERK in RAW264.7 macrophages (Figure 3). These results suggest that TRAF6 is critical in TRAIL-induced activation of MAP kinases in osteoclastogenesis signaling.

### TRAIL-induced Activation of NFATc1 in Osteoclastogenesis is TRAF6-dependent

RANKL induces activation of the TRAF6 and c-Fos pathways, which lead to the osteoclast-specific event of autoamplification of nuclear factor of activated T cells (NFAT)c1, the master transcription factor for osteoclast differentiation [29], [30], [31]. However, it is not clear whether TRAIL induces activation of NFATc1 in osteoclastogenesis; or if TRAF6 is essential for activation of NFATc1 in TRAIL-induced osteoclast differentiation. For further support TRAIL induces osteoclast differentiation through direct engagement with TRAIL receptor and to transduce signaling via TRAF6, we used the TRAIL receptor (TRAIL-R) siRNA to knock down the expression of TRAIL-R and also used the anti-TRAIL-R antagonist Ab (R&D System, Inc., Minneapolis, MN) to neutralize the interaction between TRAIL and its receptor. The results in Figure 4 demonstrated that TRAIL-induced activation of NFATc1 was significantly inhibited by transfection of TRAF6 siRNA, TRAIL-R siRNA, or treatment with anti-TRAIL-R antagonist Ab, indicating that TRAIL-induced NFATc1 activation and osteoclast differentiation activity is dependent on TRAF6 associated signaling (Figure 4).

In addition,TRAF6 is a RING-dependent ubiquitin (Ub) ligase that in conjunction with Ubc13/Uev1A catalyzes its own auto-ubiquitination via Lys63-linked poly-Ub chains [32]. It has been demonstrated that TRAF6 RING-dependent Ub ligase activity and TRAF6 auto-ubiquitination is critical for RANKL signaling and osteoclast differentiation [32], [33], [34], [35]. It is not clear whether TRAIL induces activation of TRAF6 auto-ubiquitination and its role in osteoclastogenesis. In Figure 4, our results demonstrated that in addition to induction of NFATc1, treatment of TRAIL in RAW264.7 cells also induced ubiquitination of TRAF6, and which was significantly inhibited by transfection of TRAF6 siRNA, TRAIL-R siRNA, or treatment with anti-TRAIL-R antagonist Ab, indicating that TRAIL-induced ubiquitination of TRAF6 is involved in NFATc1 activation and osteoclast differentiation, and the ubiquitination is TRAF6-dependent.

### TRAIL-induced Osteoclast Differentiation was Completely Abolished in TRAF6 Knock Out Bone Marrow Macrophages

For further determination role of TRAF6-dependent signaling in TRAIL-induced osteoclast differentiation, we isolated bone marrow derived macrophage from TRAF6 knockout mice, and assayed the ability of osteoclast differentiation induced by TRAIL. The results in Figure 5 demonstrated that the TRAIL- and RANKL-induced osteoclast differentiation activity was completely abolished in TRAF6 knockout bone marrow macrophages. Our results indicated TRAF6 is essential for TRAIL-induced osteoclast differentiation.

## Discussion

In this study, we demonstrated that TRAIL induced monocyte/macrophage lineage precursor cells to differentiate into osteoclast-like cells, and TRAIL induces osteoclastic differentiation via a TRAF-6 dependent signaling pathway. Osteoclast formation from mononuclear phagocyte precursors involves interactions between TNF ligand superfamily members and their receptors [2], [3], [4], [6], [7]. However, the signaling pathways and whether TRAF6-dependent signaling is essential for TRAIL-induced osteoclast differentiation is still not clear. Our data is the first to demonstrate TRAF6 is essential for TRAIL induces osteoclast differentiation and bone resorption activity. A model for TRAIL-induced activation of the TRAF6 associated signaling pathway in osteolcast differentiation is shown in Figure 6. Upon receptor engagement, in addition to transduce apoptosis signaling pathway, TRAIL induces activation of TRAF6 via a distinct signaling pathway. TRAF6 is the key molecule linking cytoplasmic signaling to the nuclear transcriptional program in osteoclast differentiation and bone resorption. TNF superfamily molecules other than RANKL may modify RANK signaling by interaction with TRAF6-associated signaling. This will provide new insights into the molecular mechanism which may implicate osteoimmunology in the immune response associated with bone absorption.

The present observation that TRAIL can enhance osteoclast differentiation from monocyte lineages through activation of TRAF6 and NFATc1 further strengthens the significance of distinct signaling to modulate cell function in addition to inducing apoptosis. Our results further indicate that TRAIL induces osteoclast differentiation via signaling pathways dependent on TRAF6. TRAF6 is perhaps the most important RANK-associated molecule regulating osteoclast formation and function. The significance of TRAF6 in the maintenance of normal bone architecture was demonstrated in TRAF6 knockout mice, which displayed a defect in NF-κB signaling and consequently developed osteopetrosis [16], [17]. Although their ultimate consequences are often transcriptional, the osteoclast-regulating signals directly mediated by TRAF6, identified to date, occur in the cytoplasm. These signals include activation of NF-κB by a process of ubiquitination in which TRAF6 functions as an E3 ligase [36]. Our results support the notion that TRAF6 is the key molecule linking cytoplasmic signaling to the nuclear transcriptional program in osteoclast differentiation and bone resorption. This study suggests that TRAF6-dependent signaling may be a central pathway in osteoclast differentiation, and TNFs other than RANKL may modify RANK signaling by interaction with TRAF6-associated signaling.

RANKL induces activation of the TRAF6 and c-Fos pathways, which lead to the osteoclast-specific event, that is, autoamplification of NFATc1, the master transcription factor for osteoclast differentiation [29], [30], [31]. Our results demonstrated that in TRAIL induces activation of NFATc1 in osteoclast differentiation, and knockdown of TRAF6 abolished the NFATc1 activity induced by TRAIL (Figure 4), indicating that TRAIL- induced NFATc1activation and osteoclast differentiation activity is dependent on TRAF6 associated signaling.

In conclusion, our results demonstrated that TRAF6 is essential for TRAIL induced osteoclast differentiation and bone resorption activity. Our study supports the notion that TNF superfamily molecules other than RANKL may modify RANK signaling by interaction with TRAF6-associated signaling. The novel function of TRAF6 in TRAIL-mediated osteoclastogenesis demonstrated in this study indicates a new role for TRAIL in regulating osteoclastogenesis, and will be helpful in developing better strategies for treating inflammation-induced bone resorption in the future.

## Supporting Information

Figure S1 **TRAIL-induced formation of osteoclast-like multinucleated cells from human monocytes.** (A). Human peripheral blood mononuclear cells (PBMCs) were plated in 96-well plates at 1.5×10^5^ cells/well, and the next day the adherent monocytes were treated with TRAIL at the concentrations indicated. After incubation, cells were subjected to the TRAP assay. Cell morphology was examined by light microscopy. (B). The adherent human monocytes were treated with TRAIL at the concentrations indicated, and were subjected to dell death assay. The cell death was analyzed with MTT cell viability assay. The Etoposide serves as a positive control for cell death.
(TIF)
