# Supplementary figures and images for: TRAF-6 Dependent Signaling Pathway Is Essential for TNF-Related Apoptosis-Inducing Ligand (TRAIL) Induces Osteoclast Differentiation
Source: PLoS One. 2012 Jun 14;7(6):e38048. doi: 10.1371/journal.pone.0038048 (PMC3375273; doi:10.1371/journal.pone.0038048)

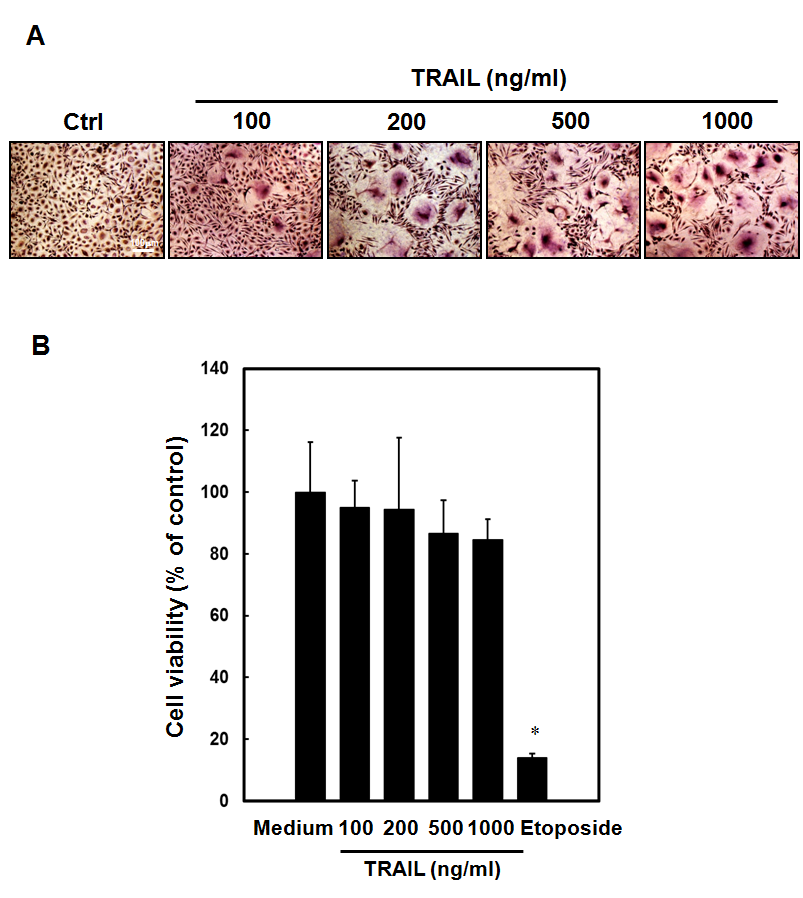

Supplement: Figure S1 — TRAIL-induced formation of osteoclast-like multinucleated cells from human monocytes. (A). Human peripheral blood mononuclear cells (PBMCs) were plated in 96-well plates at 1.5×10^5^ cells/well, and the next day the adherent monocytes were treated with TRAIL at the concentrations indicated. After incubation, cells were subjected to the TRAP assay. Cell morphology was examined by light microscopy. (B). The adherent human monocytes were treated with TRAIL at the concentrations indicated, and were subjected to dell death assay. The cell death was analyzed with MTT cell viability assay. The Etoposide serves as a positive control for cell death. (TIF) [file pone.0038048.s001.tif]
